# Targeting multidrug-resistant ovarian cancer through estrogen receptor α dependent ATP depletion caused by hyperactivation of the unfolded protein response

**DOI:** 10.18632/oncotarget.10819

**Published:** 2016-07-24

**Authors:** Xiaobin Zheng, Neal Andruska, Michael J. Lambrecht, Sisi He, Amadeo Parissenti, Paul J. Hergenrother, Erik R. Nelson, David J. Shapiro

**Affiliations:** ^1^ Department of Biochemistry University of Illinois, Urbana, IL, USA; ^2^ Department of Chemistry, University of Illinois, Urbana, IL, USA; ^3^ Department of Molecular Integrative Physiology, University of Illinois, Urbana, IL, USA; ^4^ Cancer Research Program, Advanced Medical Research Institute of Canada, Sudbury, ON, Canada; ^5^ University of Illinois Cancer Center, Urbana, IL, USA; ^6^ College of Medicine, University of Illinois, Urbana, IL, USA

**Keywords:** ERα biomodulator, ATP depletion, unfolded protein response, MDR1/P-glycoprotein/ABCB1, OVCAR-3 ovarian cancer

## Abstract

Ovarian cancers often recur and tumors acquire resistance to chemotherapy due to overexpression of the ATP-dependent efflux pump, multidrug resistance protein 1 (MDR1/P-glycoprotein/ABCB1). Nontoxic small molecule inhibitors targeting MDR1 have remained largely elusive. Instead, in a novel application of our recently described estrogen receptor α (ERα) biomodulator, BHPI, we targeted MDR1’s substrate, ATP. BHPI depletes intracellular ATP and nearly blocks MDR1-mediated drug efflux in ovarian cancer cells by inducing toxic hyperactivation of the endoplasmic reticulum stress sensor, the unfolded protein response (UPR). BHPI increased sensitivity of MDR1 overexpressing multidrug resistant OVCAR-3 ovarian cancer cells to killing by paclitaxel by >1,000 fold. BHPI also restored doxorubicin sensitivity in OVCAR-3 cells and in MDR1 overexpressing breast cancer cells. In an orthotopic OVCAR-3 xenograft model, paclitaxel was ineffective and the paclitaxel-treated group was uniquely prone to form large secondary tumors in adjacent tissue. BHPI alone strongly reduced tumor growth. Notably, tumors were undetectable in mice treated with BHPI plus paclitaxel. Compared to control ovarian tumors, after the combination therapy, levels of the plasma ovarian cancer biomarker CA125 were at least several hundred folds lower; moreover, CA125 levels progressively declined to undetectable. Targeting MDR1 through UPR-dependent ATP depletion represents a promising therapeutic strategy.

## INTRODUCTION

Ovarian cancer usually presents at an advanced stage and more than half of ovarian cancer patients die within 5 years [1-3]. Although 30-70% of ovarian tumors are estrogen receptor α (ERα) positive, endocrine therapy is largely ineffective [4-6]. Recurrent ovarian tumors are therefore treated with chemotherapy. Although initially responsive, after several cycles of treatment tumors often recur as resistant ovarian cancer, with few therapeutic options [7]. In ovarian cancer, the most common mechanism for resistance to paclitaxel and other chemotherapeutic agents is overexpression of ATP-dependent membrane efflux pumps of the ABC transporter family, especially Multidrug Resistance Protein 1 (MDR1)/P-glycoprotein/ABCB1 [8-13]. MDR1-mediated efflux reduces intracellular drug concentrations to levels at which the drugs are no longer effective at doses patients can tolerate [8, 12, 13]. Despite intensive efforts, clinically effective non-toxic small molecule MDR1 inhibitors have not been described [14]. Instead of inhibition of MDR1 we target its substrate, ATP. MDR1-mediated efflux is exquisitely sensitive to reductions in ATP levels [15-17]. However, selective depletion of ATP in cancer cells has been little studied and is difficult to achieve.

We recently described the novel non-competitive estrogen receptor α (ERα) biomodulator, BHPI, which is effective in models of ERα^+^ breast cancer [18]. In cancer cells, BHPI, acting *via* ERα, induces sustained toxic hyperactivation of the endoplasmic reticulum (EnR) stress sensor, the unfolded protein response (UPR) [18]. The UPR consists of three main branches that together balance the synthesis of new proteins with the availability of chaperones and other proteins to help fold and transport proteins within cells [19, 20]. In the classical reactive mode, EnR stress resulting from accumulation of unfolded or misfolded protein, or other stresses, triggers UPR activation [19-21]. In the recently unveiled anticipatory mode of UPR activation, estrogen or other mitogenic hormones pre-activate the UPR and anticipate a future need for increased protein folding capacity [22, 23]. BHPI distorts this normal anticipatory pathway by binding to a different site on ERα than estrogens and inducing a different ERα conformation [18]. This enables BHPI to act through ERα to hyperactivate the UPR, converting it from protective to toxic [18]. BHPI strongly activates phospholipase C γ (PLCγ), producing inositol triphosphate (IP_3_), which binds to and opens endoplasmic reticulum IP_3_ Receptor (IP_3_R) calcium channels allowing rapid efflux of calcium from the lumen of the EnR into the cytosol.

Intracellular calcium levels are tightly regulated by EnR transport channels and pumps [24, 25]. Opening the IP_3_Rs and ryanodine receptor (RyR) calcium channels allows efflux of the high concentrations of Ca^2+^ stored in the lumen of the EnR into the cytosol [26-28]. To produce this concentration gradient, powerful ATP-dependent sarcoplasmic/endoplasmic reticulum calcium-ATPase (SERCA) pumps in the EnR membrane pump calcium from the cytosol into the EnR lumen [29-31]. We show that BHPI elicits a sustained, IP_3_R dependent, increase in cytosol calcium in ovarian cancer cells. Since the IP_3_R calcium channels remain open after BHPI treatment, the calcium pumped into the EnR by the ATP-dependent SERCA pumps rapidly leaks back out. We hypothesized that sustained BHPI hyperactivation of the UPR creates a futile cycle depleting intracellular ATP, and this ATP depletion might provide a novel way to inactivate MDR1.

Using cell-based and *in vivo* studies we evaluated the potential of this novel approach to restoring chemosensitivity of multidrug resistant ovarian tumors. Notably, in OVCAR-3 ovarian cancer cells, which are resistant to micromolar paclitaxel, BHPI restored sensitivity to therapeutically relevant low nanomolar concentrations of paclitaxel. We preformed what is perhaps the first orthotopic intra-ovarian mouse xenograft study using multidrug resistant OVCAR-3 cells. Surprisingly, paclitaxel was both ineffective and actually appeared to promote metastases, a result not seen in the other treatment groups. Notably, no ovarian tumors were detected in any of the mice treated with BHPI plus paclitaxel. Moreover, levels of the circulating ovarian cancer marker, CA125/mucin 16, declined from ∼700 units/ml in control vehicle-treated mice to undetectable in all of the BHPI plus paclitaxel treated mice.

## RESULTS

### BHPI induces a sustained increase in intracellular calcium through activation of the ERα-PLCγ-IP_3_R pathway

**Figure 1:**
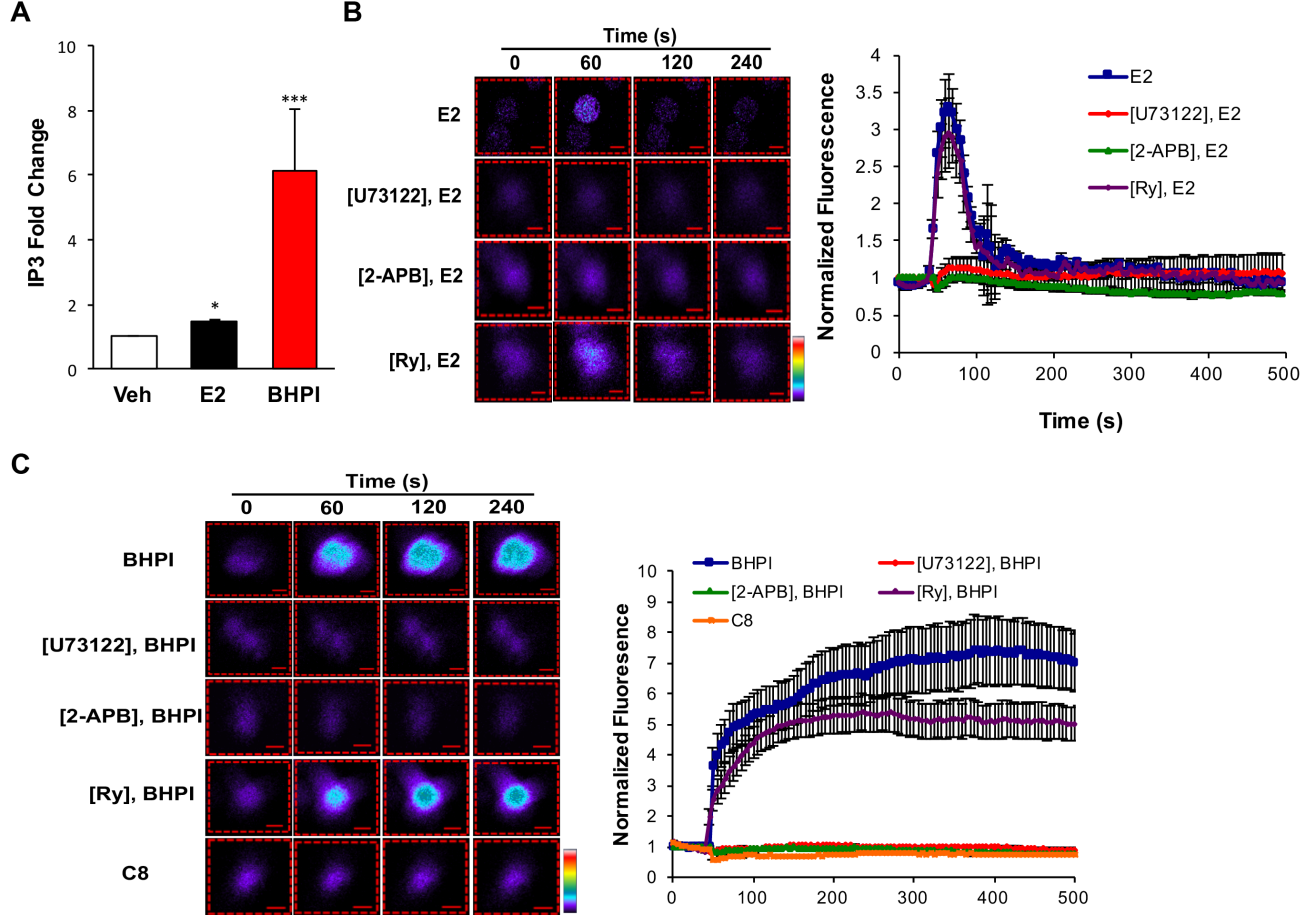
BHPI and estrogen stimulate release of calcium from the endoplasmic reticulum into the cytosol **A.** Quantitation of intracellular IP_3_ levels after 10 min treatment with DMSO, 17β-estradiol (E_2_), or BHPI in PEO-4 ovarian cells (*n* = 3). **B.**, **C.** Estrogen and BHPI increase cytosol calcium levels. Visualization of cytosolic Ca^2+^ using Fluo-4 AM; estrogen or BHPI was added to PEO-4 cells at 50 s. Color scale from basal Ca^2+^ to highest Ca^2+^: purple, blue, green, yellow, red, white. Quantitation of cytosolic Ca^2+^ levels after pre-treating PEO-4 cells with U73122, 2-APB, or Ry followed by treatment with estrogen or BHPI (*n* = 12-20). Concentrations: U73122, 1 μM; 2-APB, 100 μM; Ry, C8, 10 μM; E_2_, 200 nM; BHPI, 10 μM. *P < 0.05, ***P < 0.001.

Using breast cancer cells, we previously showed E_2_-ERα activates a PLCγ-IP_3_R pathway to release calcium from EnR stores into the cytosol [32]. Activated PLCγ cleaves its substrate to produce IP_3_. The non-competitive ERα biomodulator BHPI, that works by hyperactivating the UPR, produces much higher levels of IP_3_ than E_2_ [18]. If we were to use this pathway to target multidrug resistant ovarian cancer, we had to first show that the pathway functions in ovarian cancer cells. We initially quantitated IP_3_ levels in ERα^+^ PEO-4 ovarian cancer cells treated with E_2_ or BHPI. E_2_ induced a modest increase in IP_3_ levels, while BHPI induced a much more robust 6-fold increase (Figure 1A).

To test whether E_2_ and BHPI rapidly increase cytosolic Ca^2+^, we monitored calcium levels using the fluorescent calcium sensor dye Fluo-4 AM. In < 1 min., E_2_ and BHPI increased cytosol Ca^2+^ in PEO-4 cells (Figure 1B, 1C). Notably, in the absence of extracellular calcium, E_2_ elicited a transient ∼3.5 fold increase in cytosolic Ca^2+^ with the Ca^2+^ signal rapidly returning to the basal level (Figure 1B and Supplementary Movie 1). In contrast, BHPI elicited a sustained ∼7 fold increase in cytosolic Ca^2+^ (Figure 1C and Supplementary Movie 2). Since pretreatment with the PLCγ inhibitor U73122 abolished the calcium release observed with E_2_ or BHPI, PLCγ activation was required for the increase in cytosolic Ca^2+^ (Figure 1B, 1C and Supplementary Figure 1). BHPI induced a large increase in cytosolic Ca^2+^ even in the absence of extracellular Ca^2+^, indicating that BHPI increases cytosolic Ca^2+^ by depleting the Ca^2+^ store in the EnR. Supporting this idea is our observation that inhibiting the IP_3_R Ca^2+^ channel with 2-APB abolished the rapid E_2_ and BHPI stimulated Ca^2+^ release (Figure 1B, 1C and Supplementary Figure 1). In contrast, inhibition of RyR calcium channels with high concentration ryanodine (Ry) did not block E_2_ or BHPI stimulated Ca^2+^ release in PEO-4 cells (Figure 1B, 1C). Confirming BHPI’s structural specificity and the requirement for ERα, a control compound, C8, that is structurally related to BHPI, but does not bind ERα *in vitro* [18], failed to increase cytosolic Ca^2+^ (Figure 1C). These results demonstrate that BHPI strongly activates the ERα-PLCγ-IP_3_R pathway in ovarian cancer cells, resulting in a sustained increase in cytosolic Ca^2+^.

### BHPI activates the UPR in ovarian cancer cells

**Figure 2:**
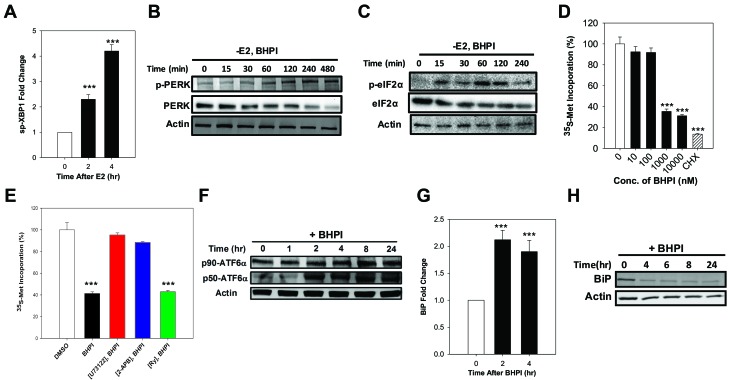
In PEO-4 ovarian cells, BHPI activates the three branches of the UPR and inhibits protein synthesis **A.** qRT-PCR analysis showing the increased level of spliced XBP1 (sp-XBP1) mRNA (*n* = 3). **B.**, **C.** Western blot analysis showing time dependent phosphorylation of PERK and eIF2α. **D.** Protein synthesis after treating the cells with increasing concentrations of BHPI (*n* = 4). CHX, cycloheximide. Protein synthesis from DMSO treated control cells was set to 100%. **E.** The level of protein synthesis after pretreating the cells with either the inhibitors U73122, 2-APB, or Ry followed by BHPI treatment (*n* = 4). **F.** Western blot analysis shows full-length (p90-ATF6α) and cleaved p50-ATF6α in BHPI treated cells. Effect of BHPI on the level of BiP mRNA level **G.** and protein **H.**. Concentrations: U73122, 1 μM; 2-APB, 100 μM; BHPI, 500 nM (E) or 1 μM (other panels). ***P < 0.001.

Efflux of calcium stored in the lumen of the EnR into the cell body activates the UPR. The core UPR signaling cascade consists of 3 EnR sensors whose activation increases protein-folding capacity and temporarily reduces protein production (Supplementary Figure 1). Activation of IRE1α, which alternatively splices the transcription factor XBP1, produces the widely used UPR marker, active spliced XBP1 (sp-XBP1) [33]. Supporting activation of the IRE1α branch of the UPR, in PEO-4 ovarian cancer cells, BHPI and E_2_ robustly induced sp-XBP1 (Figure 2A and Supplementary Figure 2A). Protein synthesis is regulated by autophosphorylation of PERK [34]. Phosphorylated PERK (p-PERK) phosphorylates of eukaryotic initiation factor 2 α (eIF2α), which leads to transient inhibition of protein synthesis (Supplementary Figure 1). E_2_ induced a weak and transient phosphorylation of eIF2α in ovarian cells (Supplementary Figure 2B), while BHPI elicited robust phosphorylation of PERK and eIF2α (Figure 2B, 2C), resulting in inhibition of most protein synthesis (Figure 2D) and a decline in total PERK and eIF2α protein (Figure 2B, 2C). Consistent with their inhibitors’ ability to block calcium efflux (Figure 1B, 1C), inhibition of PLCγ with U73122 and locking the IP_3_R calcium channels with 2-APB, but not inhibition of the RyR calcium channels, reversed BHPI inhibition of protein synthesis (Figure 2E). EnR stress leads to proteolytic cleavage of ATF6α to active 50 kDa ATF6α (p50-ATF6α) (Supplementary Figure 1) [35]. Demonstrating BHPI and E_2_ activate the ATF6α arm of UPR, E_2_ and BHPI increased p50-ATF6α levels in PEO-4 cells (Figure 2F and Supplementary Figure 2C). Active p50-ATF6α increases production of BiP/GRP78/HSPA5 and other EnR chaperones [35]. BHPI and E_2_ increased production of BiP mRNA in PEO-4 ovarian cancer cells (Figure 2G and Supplementary Figure 2D). However, since BHPI inhibited protein synthesis (Figure 2D and 2E), BiP protein levels were reduced in BHPI-treated PEO-4 cells (Figure 2H).

Collectively, our findings in ovarian cancer cells indicate that E_2_-ERα induces weak and transient anticipatory activation of the UPR and that BHPI distorts this UPR pathway resulting in strong and sustained UPR activation. These data provide a potential mechanism for inactivating MDR1 in ovarian cancer cells.

### BHPI depletes intracellular ATP inactivating MDR1-mediated efflux

**Figure 3:**
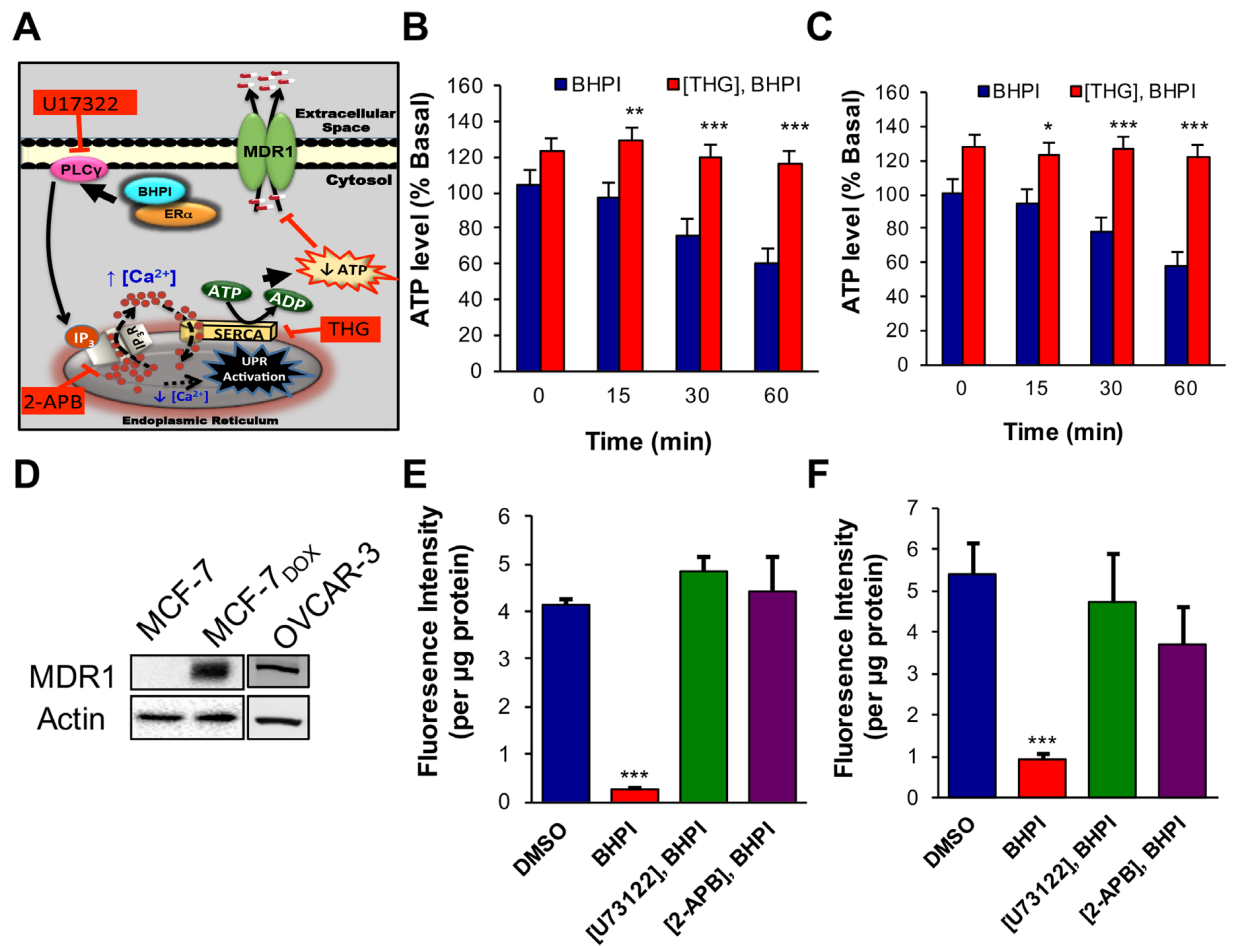
In MDR1 overexpressing cells, BHPI depletes intracellular ATP and inhibits MDR1 efflux activity **A.** Proposed model of the pathway by which BHPI inactivates MDR1. **B.**, **C.** Intracellular ATP quantitation showing effect of BHPI on cellular ATP level after pretreating cells with either DMSO or thapsigargin (THG) (*n* = 6). **D.** Western blot analysis showing MDR1 protein level from the indicated cell lines. **E.**, **F.** Rhodamine-123 (Rho-123) quantitation showing the effect of BHPI on Rho-123 concentration in the media after pretreating cells with either DMSO, U73122, or 2-APB (*n* = 6). Concentrations: U73122, 1 μM; 2-APB, 100 μM; BHPI, 1 μM (B, C) or 500 nM (E, F). Data is the mean ± SEM. ***P < 0.001.

We hypothesize: (i) In response to the BHPI-mediated loss of EnR calcium, SERCA pumps will carry out ATP-dependent transport of Ca^2+^ from the cytosol back into the lumen of the EnR. (ii) Since BHPI elicits sustained increases in cytosolic Ca^2+^ (Figure 1C, Video 2), indicating the IP_3_R calcium channels remain open, calcium pumped from the cytosol into the lumen of the EnR leaks back out through the open IP_3_R channels, creating a futile cycle that depletes ATP (Figure 3A). To test our hypothesis we investigated the effect of BHPI on ATP levels in ovarian cancer cells. BHPI treatment rapidly reduced intracellular ATP levels in ERα^+^ PEO-4 and OVCAR-3 ovarian cancer cells (Figure 3B, 3C). Supporting the role of the EnR SERCA pumps in ATP depletion, the SERCA pump inhibitor, thapsigargin (THG) blocked the decline in ATP levels seen after BHPI treatment (Figure 3B, 3C).

ERα^+^, multidrug resistant, OVCAR-3 ovarian cancer cells were derived from a patient whose cancer recurred after surgery and multiple rounds of chemotherapy [36]. OVCAR-3 cells have been propagated without cloning and therefore retain much of the diversity of a patient derived xenograft [36, 37]. ERα^+^ MCF-7 breast cancer cells are normally MDR1 negative and sensitive to the chemotherapy agent doxorubicin. MCF-7 doxorubicin (MCF-7_dox_) resistant breast cancer cells were generated by selection in increasing doxorubicin concentrations [38, 39]. Confirming that upregulation of MDR1 is a common mechanism in cancer cells resistant to cytotoxic chemotherapy, both the MCF-7_dox_ and OVCAR-3 cells overexpress MDR1 (Figure 3D). Notably, while 50 nM BHPI blocked MCF-7 proliferation, 500 nM BHPI was required to block proliferation of the MCF-7_dox_ breast cancer cells (Supplementary Figure 3A, 3B). This is consistent with the possibility that BHPI may be an MDR1 substrate.

The fluorescent MDR1 substrate Rhodamine 123 (Rho-123) is widely used to quantitate MDR1-mediated efflux from cells into the medium [14, 40]. OVCAR-3 and MCF-7_dox_ cells that overexpress MDR1, and control MDR1 negative PEO-4 cells were preloaded with Rho-123 and Rho-123 efflux into the medium was quantitated. Rho-123 efflux from the MDR1 negative PEO-4 cells was negligible (Supplementary Figure 4). OVCAR-3 and MCF-7_dox_ cells exhibited robust time-dependent efflux of Rho-123 (Supplementary Figure 4). We tested whether BHPI-treatment, which reduces intracellular ATP levels, inhibits MDR1-mediated Rho-123 efflux. In OVCAR-3 and MCF-7_Dox_ cells BHPI nearly abolished Rho-123 efflux (Figure 3E, 3F). Consistent with the proposed futile cycle leading to ATP depletion causing inhibition of MDR1-mediated efflux (Figure 3A), inhibiting the rise in intracellular Ca^2+^ by either inhibiting PLCγ with U73122, or by locking the EnR IP_3_R calcium channels closed with 2-APB (Figure 1C), reversed BHPI inhibition of MDR1-mediated efflux (Figure 3E, 3F). We next explored whether other actions of BHPI might complement ATP depletion and contribute to the near abolition of MDR1-mediated efflux.

**Figure 4:**
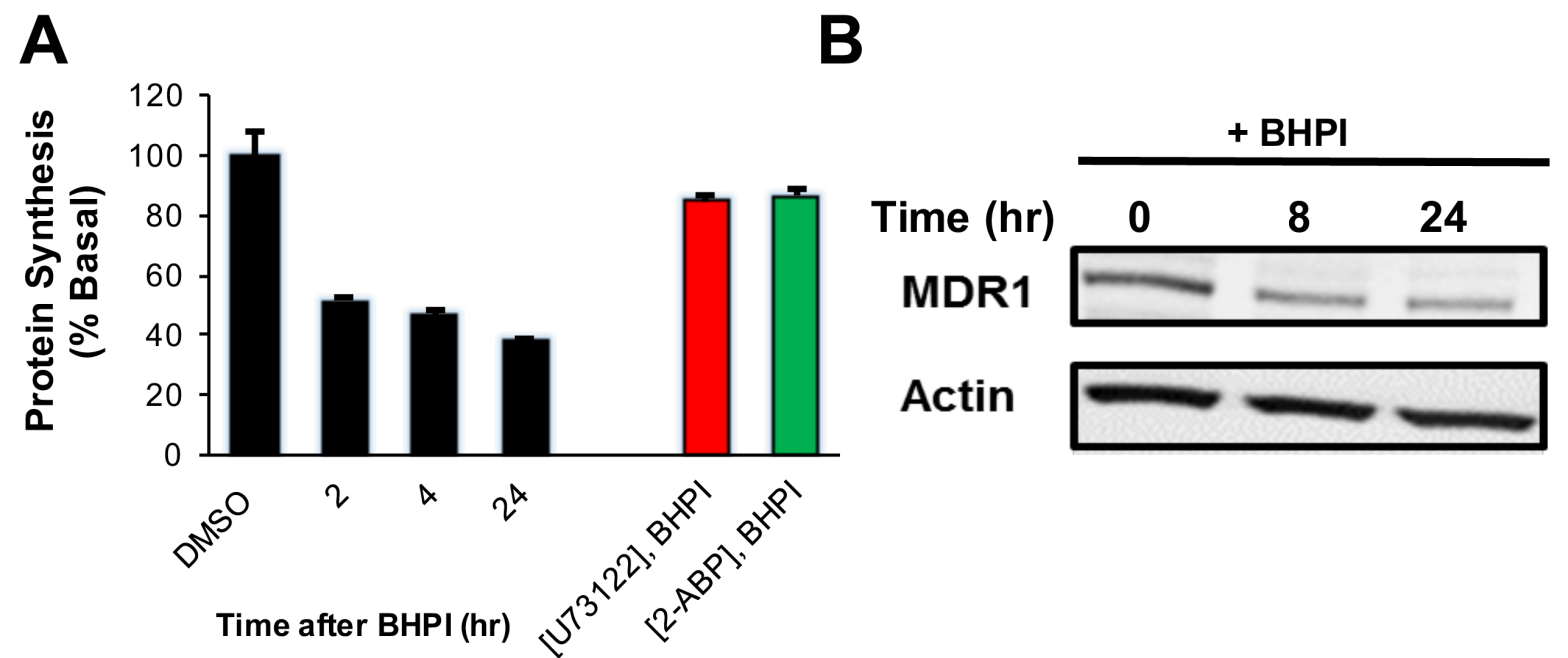
BHPI inhibits protein synthesis and reduces the level of MDR1 protein in OVCAR-3 cells **A.** Protein synthesis was measured using the incorporation of ^35^S methionine into protein at the indicated times after pretreating cells for 20 min. with either DMSO, U73122, or 2-APB (*n* = 4). **B.** Western blot indicating the MDR1 protein level at indicated time points after BHPI treatment. Concentrations: U73122, 1 μM; 2-APB, 100 μM; BHPI, 500 nM (A) or 1 μM (B) . Data is the mean ± SEM.

In OVCAR-3 cells, BHPI elicited strong and sustained activation of the PERK arm of the UPR inhibiting protein synthesis cells by ∼60% (Figure 4A). This reduced production of protein led to an ∼2 fold decline in MDR1 levels (Figure 4B). Therefore, the reduced level of MDR1 and the decline in the level of its substrate ATP, work together to enable BHPI to nearly abolish MDR1-mediated efflux. We therefore tested whether BHPI could restore sensitivity of OVCAR-3 and MCF-7_dox_ cells to therapeutically relevant concentrations of chemotherapy drugs.

### BHPI resensitizes resistant cancer cells to paclitaxel and doxorubicin

**Figure 5:**
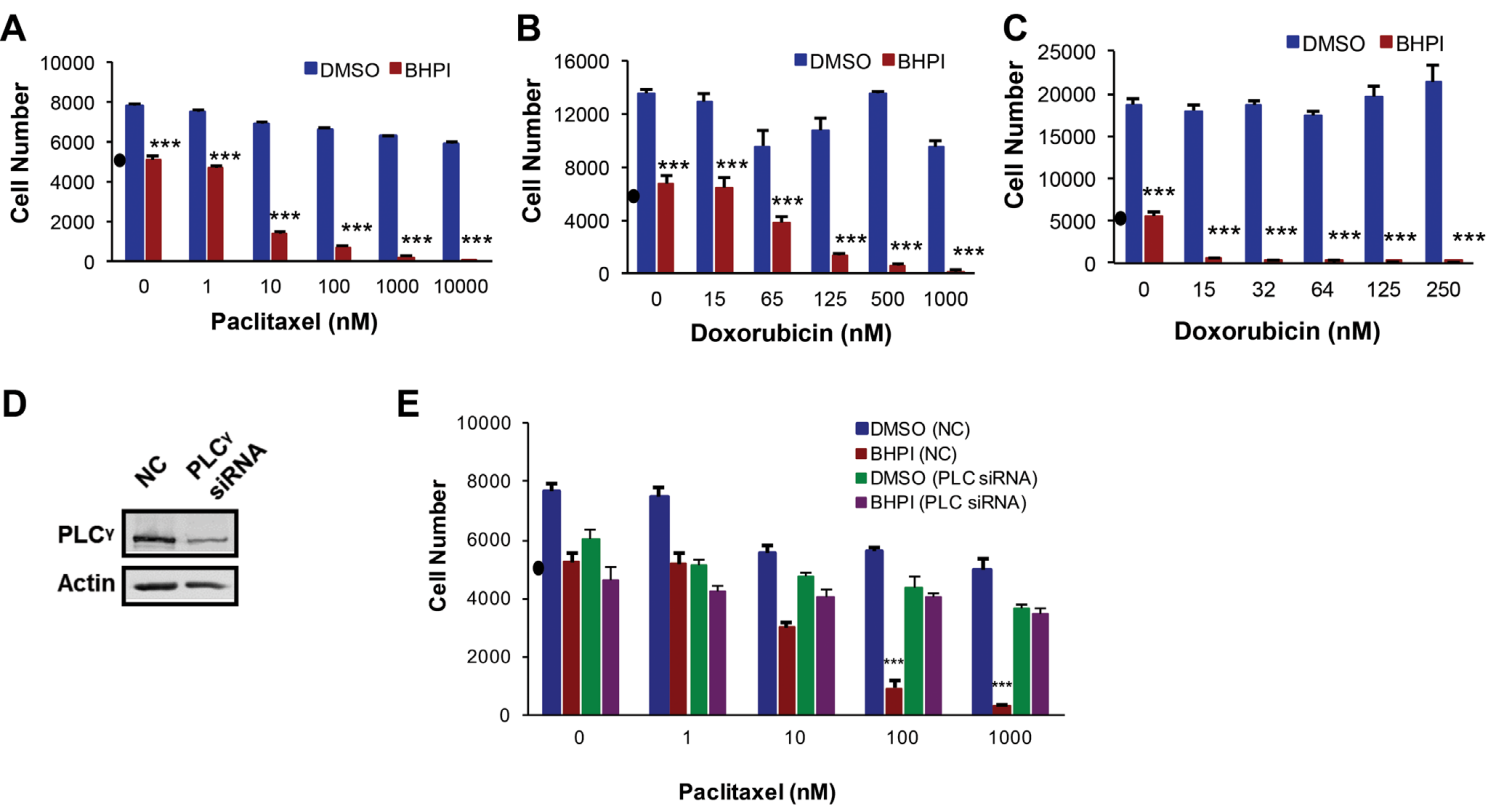
BHPI restores drug sensitivity in MDR1 overexpressing cells MTS assays showing the effect of BHPI (1 μM) plus either DMSO or the indicated concentrations of paclitaxel **A.** or doxorubicin **B.** in OVCAR-3 ovarian cells (*n* = 6) or doxorubicin in MCF-7_dox_ breast cells **C.** (*n* = 6). **D.** Western blot analysis showing the PLCγ protein level after transfecting the cells with either non-coding SmartPool siRNA or 100 nM SmartPool PLCγ siRNA. **E.** PLCγ knockdown abolishes the ability of BHPI to reverse multidrug resistance. OVCAR-3 cells were transfected with either non-coding control or PLCγ siRNA and the effect on cell number in cells treated with vehicle of BHPI was determined (*n* = 6). Cell number in a-e is from standard curves of absorbance *versus* cell number for each cell line. “•” on each graph denotes the number of cells at the start of the experiments. Data is the mean ± SEM. ***P < 0.001.

OVCAR-3 cells were highly resistant to paclitaxel, and were not killed, even at 10,000 nM paclitaxel (Figure 5A). While BHPI alone blocked OVCAR-3 cell growth, it was not cytotoxic. BHPI restored the cytotoxicity of paclitaxel at 10 nM paclitaxel, reducing the number of OVCAR-3 cells by ∼70% in two days (Figure 5A). This represents an > 1,000 fold increase in sensitivity to paclitaxel. Furthermore, OVCAR-3 cells were also resistant to 1,000 nM doxorubicin; BHPI also restored sensitivity to doxorubicin (Figure 5B). MCF-7_dox_ breast cancer cells were resistant to 250 nM doxorubicin. BHPI restores sensitivity of the MCF-7_dox_ cells, to the lowest dose of doxorubicin tested (15 nM) (Figure 5C). Importantly, since the therapeutic range of concentrations is ∼15-20 nM for paclitaxel and 100-150 nM for doxorubicin [37, 41, 42], BHPI restored sensitivity of multidrug resistant ovarian and breast cancer cells to therapeutically relevant concentrations of paclitaxel and doxorubicin.

We propose that BHPI restores drug sensitivity because it strongly activates the ERα-PLCγ-IP_3_R pathway leading to ATP depletion and a moderate reduction in MDR1 expression. A testable alternative is that the BHPI-mediated >1,000 fold increase in sensitivity of OVCAR-3 cells to killing by paclitaxel is simply due to combinatorial actions of two toxic drugs, BHPI and paclitaxel. OVCAR-3 cells are also resistant to cisplatin, which is not a substrate of MDR1 and is therefore not pumped out by MDR1 [43]. BHPI treatment did not restore sensitivity of OVCAR-3 cells to killing by cisplatin (Supplementary Figure 5A). Thus, BHPI’s ability to abolish multidrug resistance is due to its ability to interfere with MDR1, and not to the additive effects of BHPI in combination with a chemotherapeutic. Consistent with BHPI acting through ERα, BHPI did not inhibit proliferation or restore paclitaxel sensitivity in ERα negative MDR1 overexpressing NIH/ADRes ovarian cancer cells (Supplementary Figure 5B). In addition, the inactive structural relative of BHPI, C8, did not restore paclitaxel or doxorubicin sensitivity in OVCAR-3 cells (Supplementary Figure 5C).

We next sought to confirm that the ability of BHPI to resensitize OVCAR-3 cells to paclitaxel was mediated by the PLCγ pathway. Although useful in short-term studies, the long-term use of PLCγ and IP_3_R inhibitors U73122 and 2-APB may result in secondary effects. Since simultaneous knockdown of the three IP_3_R channels is somewhat toxic [18], it cannot be combined with the two other drugs. We therefore evaluated the effect of siRNA knockdown of PLCγ on paclitaxel sensitivity in BHPI-treated OVCAR-3 cells. siRNA knockdown of PLCγ, but not a control siRNA, abolished BHPI-mediated restoration of paclitaxel sensitivity (Figure 5D, 5E). Thus, BHPI’s novel mechanism of action leads to inactivation of MDR1 in multiple cell models, resulting in restoration of sensitivity to therapeutically relevant concentrations of paclitaxel and doxorubicin.

### BHPI restores paclitaxel sensitivity and eliminates tumors in a multidrug resistant ovarian tumor model

**Figure 6:**
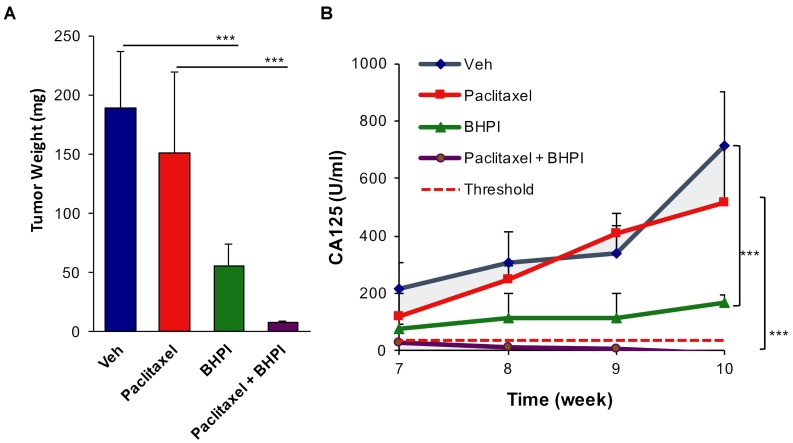
BHPI plus paclitaxel eliminates orthotopic multidrug resistant OVCAR-3 tumors **A.** Average tumor weight from each treatment group (*n* = 5). For the paclitaxel group the secondary growths were included in tumor weight. **B.** Circulating serum CA125 biomarker quantitation showing the progression of tumors in each treatment group (*n* = 5). Threshold in humans (dashed line) denotes 35 U/ml of circulating CA125. Data is the mean ± SEM. ***P < 0.001.

To assess *in vivo* effectiveness of BHPI in restoring drug sensitivity, we used OVCAR-3 cells, which are resistant to therapeutically relevant concentrations of all common anticancer drugs [36]. We used an orthotopic model in which OVCAR-3 cells were grafted into the bursa of one ovary, the other ovary serving as a control. At the end of the 10 week study, ovarian tumors were evident in each of the vehicle-treated mice, with an average weight of ∼200 mg (Figure 6A AND Supplementary Figure 6). Surprisingly, in the paclitaxel-treated group there were large secondary tumors in adjacent tissue (Supplementary Figure 6). Increased metastasis of paclitaxel-treated OVCAR-3 tumors has not been previously described because this is perhaps the first use of OVCAR-3 cells in an orthotopic ovarian model [44]. BHPI alone very significantly reduced tumor size and weight (Figure 6A and Supplementary Figure 6). Notably, there were no visible OVCAR-3 ovarian tumors in the combined BHPI and paclitaxel treatment group and no secondary tumors were detected (Figure 6A and Supplementary Figure 6). In the BHPI plus paclitaxel group, the ovary injected with OVCAR-3 cancer cells and the control ovary appeared identical.

Although tumors were not visible in the BHPI plus paclitaxel group, to more sensitively assess whether tumor cells were still present, we quantified the circulating level of serum CA125 tumor antigen. In ovarian cancer, the circulating level of CA125 is a widely used biomarker for therapeutic progress and tumor recurrence [45-47]. Although the basal level of CA125 in normal human serum is ∼35 U/ml (Figure 6B, dashed line), the human CA125 antibody does not cross-react with control mouse serum. Thus, the level of serum CA125 is a sensitive marker for the survival of human OVCAR-3 cancer cells in the mice. Serum samples were taken in weeks 7-10 of the study and assayed after completion of the study. CA125 levels in the control vehicle-treated group and in the paclitaxel-treated group increased dramatically in weeks 7-10. Confirming that the OVCAR-3 tumors are highly paclitaxel-resistant, CA125 levels were similar in the control vehicle-treated and paclitaxel-treated mice (Figure 6B). BHPI strongly reduced circulating CA125 levels compared to vehicle or paclitaxel alone, but CA125 levels rise slightly from weeks 7-10 (Figure 6B, green line). Strikingly, in the BHPI plus paclitaxel treated mice, CA125 levels declined from a low starting level of ∼30 U/ml at week 7 to concentrations below the detection limits for all five mice at week 10 (Figure 6B, purple line). Since the detection limit of the assay is ± 5 U/ml and the vehicle-treated group had circulating CA125 levels of ∼700 U/ml, tumor burden was reduced by 200 fold or more in mice to undetectable levels after combined BHPI and paclitaxel treatment. Measurement of mouse body weights throughout the study suggested that BHPI alone and BHPI plus paclitaxel were well tolerated and no visible toxic effects of BHPI were seen in the mice (Supplementary Figure 7).

## DISCUSSION

Although 30-70% of ovarian cancers are ERα^+^ at diagnosis, endocrine therapy is largely ineffective [1-3]. The failure of endocrine therapy raises the possibility that the presence or absence of ERα has little effect on ovarian tumors and there is no selection pressure to maintain ERα in recurrent multidrug resistant tumors. However, recent studies show that estrogens, acting through ERα, enhance ovarian tumor growth and increase risk of lymphovascular space invasion [48, 49]. Moreover, ERα expression correlates with poor clinical outcome in ovarian cancer [50]. The association of ERα with late-stage therapy-resistant tumors strongly suggests that ERα is maintained in many of these tumors, making them targetable with our small molecular biomodulator.

Therapeutic options are limited for patients with recurrent multidrug resistant ovarian cancer. Overexpression of MDR1 is a major resistance mechanism [8, 13, 14]. Selective non-toxic inhibitors of MDR1 have proven difficult to identify. For MDR1 inhibitors, toxicity due to inhibition of ABC transporter family members in normal cells has been a serious concern [8]. BHPI is effective because it uses a therapeutic strategy different from classic MDR1 inhibitors and most other cytotoxic chemotherapeutic drugs [8, 14]. It works by hyperactivating the UPR, a pathway that is already partially activated as a protective mechanism in tumor cells. We recently reported that elevated expression of a UPR gene signature consisting of UPR sensors and downstream targets of UPR activation is tightly correlated with therapy resistance, tumor recurrence and a poor prognosis in ERα^+^ breast cancer [32]. In contrast, the UPR is nearly off in normal healthy cells and its components are not overexpressed [32]. Consistent with this, BHPI was well tolerated in the xenograft study. While BHPI and estrogen share a common ERα-dependent pathway for UPR activation (Supplementary Figure 1), the weak estrogen-ERα activation of the UPR induces protective chaperones and is important for subsequent estrogen-ERα activation of gene expression and induction of cell proliferation [32]. Notably, BHPI binding to ERα is not competitive with estrogen binding, indicating that they bind ERα at different sites [18]. Moreover, BHPI induces conformational changes in ERα not seen with estrogen [18]. Thus, unlike estrogen, BHPI hyperactivates the UPR, leading to persistent inhibition of protein synthesis in ERα positive cancer cells [18].

Strong and sustained activation of the UPR by BHPI creates a futile cycle leading to depletion of intracellular ATP and inactivation of MDR1-mediated efflux (Figure 3A). Supporting the proposed pathway is our observation that BHPI is only effective in ERα^+^ cells. Furthermore, inhibitor and knockdown studies demonstrate the critical roles of PLCγ, IP_3_R calcium channels and SERCA pumps. BHPI-ERα hyperactivation of the UPR results in rapid depletion of ATP leading to activation of AMPK [18]. Activated AMPK reportedly inhibits MDR1 gene expression [51, 52]. Since together the potential AMPK-mediated reduction in MDR1 gene expression and the UPR mediated inhibition of protein synthesis only reduce MDR1 protein levels ∼2 fold, they are likely to be complementary, rather than central, to the dramatic and rapid reduction in MDR1 mediated efflux and to the restoration of drug sensitivity.

Despite MDR1’s acute sensitivity to reduction in ATP levels therapeutic reduction of ATP levels has been an elusive target. The glyceraldehyde-3-phosphate dehydrogenase inhibitor, 3-bromopyruvate inhibits glycolysis, leading to loss of ATP and MDR1 inactivation [53]. However, lack of specificity, and toxicity in normal cells, have hindered therapeutic application of 3-bromopyruvate.

Ovarian cancers originate in the fallopian tubes or ovaries [5]. We used an orthotopic mouse xenograft model in which OVCAR-3 cells were grafted into the bursa of one ovary. Because these internal tumors cannot be directly measured until the study ends, serum levels of CA125 over the last 4 weeks of the study provide a surrogate marker for tumor progression. Serum CA125 levels in the paclitaxel and vehicle-treated mice increased rapidly in weeks 7-10. Tumor weight and CA125 levels indicated that the overall tumor burden was similar in the paclitaxel and vehicle-treated mice. Although the primary ovarian tumors were small in the paclitaxel-treated mice, these tumor-harboring mice were prone to developing large secondary (extra-ovarian) growths. Interestingly, increased metastasis following therapy has also been reported in prostate cancer xenografts treated with abiraterone [54] and breast cancer xenografts treated with sunitinib or bevacizumab [55]. CA125 levels and tumor weight were reduced 60-80% in the BHPI treated mice. The slight increase in CA125 levels in week 7-10 suggests BHPI strongly inhibited, but did not completely block, tumor progression. In contrast, in the BHPI plus paclitaxel treatment group, the already extremely low levels of CA125 at week 7 declined progressively to undetectable levels at week 10. This suggests ongoing tumor regression in this treatment group during the last 4 weeks of the study. Although there was substantial individual variation in tumor size and weight, and in CA125 levels, in the combined treatment group, both tumors and plasma CA125 were undetectable in all 5 mice. Absence of visible tumors, or complete loss of circulating tumor markers, has not been reported in other xenograft studies using highly drug-resistant OVCAR-3 ovarian cells [53, 56, 57].

*De novo* and acquired multidrug resistance is a core problem in cancer chemotherapy. In ovarian cancer, the primary driver of multidrug resistance is overexpression of MDR1. BHPI alone has emerged as a promising and well-tolerated therapeutic candidate for multidrug resistant ovarian cancer. Central to BHPI’s therapeutic potential is its novel mechanism of action based on strong and sustained hyperactivation of the anticipatory UPR pathway, resulting in ATP depletion and MDR1 inactivation. This enables BHPI to resensitize multidrug resistant tumors to chemotherapeutic intervention and reduce ovarian tumor burden to undetectable levels. Thus, BHPI is a unique candidate for further mechanistic exploration and therapeutic development.

## MATERIALS AND METHODS

### Cell culture and reagents

Cell culture medium and conditions were as previously described [18, 32]. Dr. S. Kaufmann provided PEO-4 ovarian cells. Dr. A. Parissenti provided MCF-7 and doxorubicin resistant MCF-7 (MCF-7_dox_) breast cancer cells. OVCAR-3 cells were obtained from the ATCC. E_2_, U73122, Rhodamine-123 (Rho-123) dye and 2-amino propyl-benzoate (2-APB) were from Sigma Aldrich (St Louis, MO, USA). Ryanodine (Ry) was from Santa Cruz Biotechnology (Danvers, MA, USA). BHPI was synthesized on gram scale *via* a short sequence. Detailed experimental protocols are available in supplementary materials.

### Western blot

Western blotting was carried out as previously described [18, 32, 58]. The following antibodies were used: Phospho-eIF2α (Ser51) (#3398; Cell Signaling Technology), eIF2α (#5324; Cell Signaling Technologies, MA), Phospho-PERK (#3179; Cell Signaling Technology, MA), PERK (#5683; Cell Signaling Technology, MA), ATF6α (Imgenex, CA), PLCγ (#5690; Cell Signaling Technology, MA), BiP (#3177; Cell Signaling Technology, MA), MDR1/ABCB1 (#12683; Cell Signaling Technology, MA) and β-Actin (Sigma, MO). The protein and antibody complexes were detected using horseradish peroxidase-conjugated secondary antibodies and chemiluminescent immunodetection with an ECL Detection Kit (GE Healthcare, NJ), and were visualized using a PhosphorImager.

### Cell proliferation assays

Cells were plated in growth media with 10% CD-FBS for three days. Subsequently, cells were resuspended in growth media with 10% CD-calf serum and plated in 96 well plates. The medium was replaced with treatment media the following day, and plates were incubated at 37°C in 5% CO_2_ for 2-4 days. During experiments, the medium was replaced every two days. Cell number was determined using MTS and CellTiter 96 Aqueous One Solution Reagent (Promega). For each cell line, cell number was calculated from a standard curve of the number of plated cells at A_490_.

### Assaying MDR1 efflux activity

Cells were plated in 6-well plates and allowed to reach 80% confluence. Subsequently, cells were loaded with 10 μM of Rho-123 (1% methanol, HBSS) for 10 minutes at 37°C. Then, cells were washed three times with cold PBS to remove residual Rho-123 and efflux started by addition of pre-warmed HBSS buffer. At the end of the measured time points, cells were lysed with 2% (v/v) triton-x100 in HBSS with proteinase inhibitor cocktail. Total protein was determined and Rho-123 concentration was normalized to total protein.

### Mouse xenografts

All experiments were approved by the Institutional Animal Care Committee (IACUC) of the University of Illinois at Urbana-Champaign. The immunodeficient NSG mice (Jackson Laboratory) were obtained from in-house breeding. 1 million OVCAR-3 cells were orthotopically grafted into the bursa of one ovary. Subsequently, the mice were randomly divided into four treatment groups. Starting one week after injecting the tumor cells each group received vehicle plus vehicle, paclitaxel plus vehicle, vehicle plus BHPI, or paclitaxel plus BHPI. The paclitaxel was dissolved in Polysorbate-80 and ethanol (1:1, vol/vol) and further diluted with saline to reach final concentration. Stock BHPI was dissolved in DMSO and further diluted with 10% Tween-20 with 88% PBS to working concentration. Doses were 10 mg/kg of paclitaxel IP injected every other day, and 50mg/kg of BHPI injected IP daily.

### Measuring levels of serum CA125

Plasma CA125 concentrations were determined by ELISA according to the manufacturer’s protocol (#KA0205; Abnova, CA). The final serum CA125 concentrations were calculated based on a standard curve.

### qRT-PCR, IP_3_ quantitation, PLCγ siRNA knockdown, calcium imaging, and protein synthesis measurements

Carried out as we recently described [18, 32, 59].

### Statistical analysis

R was used for the statistical analysis. For terminal tumor weights, one-way ANOVA followed by the Kruskal-Wallis *post hoc* test was used (*P* < 0.05). For CA125 serum analysis, two-way ANOVA followed by Bonferroni’s *post hoc t*-test was used (*P* < 0.05). Other analyses were conducted either with two-tailed Student t tests or with one-way ANOVA followed by Tukey *post hoc* tests (*P* < 0.05). Data are reported as mean ± SEM.

## SUPPLEMENTARY MATERIALS FIGURES AND VIDEOS






