# Supplementary material for: Targeting multidrug-resistant ovarian cancer through estrogen receptor α dependent ATP depletion caused by hyperactivation of the unfolded protein response
Source: Oncotarget. 2016 Jul 24;9(19):14741–53. doi: 10.18632/oncotarget.10819 (PMC5871075; doi:10.18632/oncotarget.10819)
Supplement: Supplementary file 1 [file oncotarget-09-14741-s001.pdf]

Targeting multidrug-resistant ovarian cancer through estrogen receptor α dependent ATP depletion caused by hyperactivation of the unfolded protein response

SUPPLEMENTARY MATERIAL

PROCEDURES FOR BHPI SYNTHESIS

Unless otherwise noted, all reactions were run in oven or flame-dried glassware under an atmosphere of dry nitrogen. Methylene chloride and acetonitrile were obtained from a solvent dispensing system. All other chemicals were used as received from commercial vendors. Thin-layer chromatography analysis was performed on EMD Merck silica gel plates with F254 indicator. TLC plates were visualized by UV light (254 nm) or by staining with p-anisaldehyde. Column chromatography was performed with silica gel purchased from Sorbent Technologies (40-75 μm particle size).

^{1}H and ^{13}C NMR spectra were recorded at 500 and 125 MHz, respectively and were referenced to the residual solvent peak. The University of Illinois Mass Spectrometry Center performed mass spectrometry analysis. LC/MS analysis was performed on an Agilent 6230 LC/MS TOF system with a 1.8 mm, 2.1x50 mm Agilent ZORBAX Eclipse Plus C18 column.

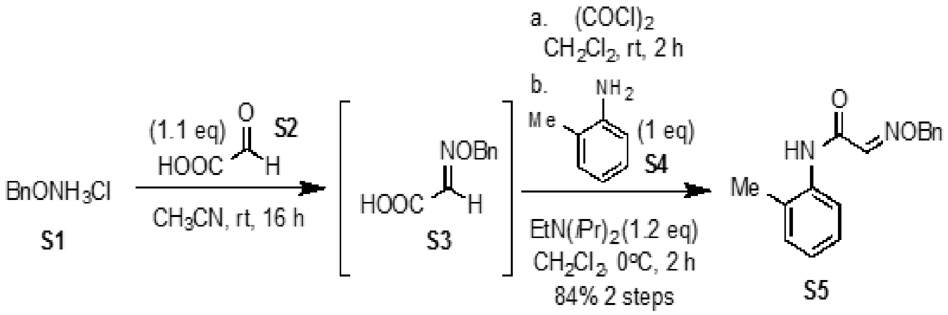

Compound S5: To a 2 L round bottom flask was added glyoxalic acid monohydrate (**S2**) (6.3 g, 68.9 mmol, 1.1 eq), *O*-Benzylhydroxylamine hydrochloride (**S1**) (10.0 g, 63.0 mmol, 1 eq), and acetonitrile (1.2 L). The reaction was stirred for 16 h at room temperature and the solvent was evaporated. The resulting white solid was dissolved in CH_2Cl_2 (640 mL) and oxalyl chloride (6.5 mL, 75.6 mmol, 1.2 eq) was added. The reaction was stirred for 2 h at room temperature and the solvent was evaporated. The resulting oil was dissolved in CH_2Cl_2 (640 mL), cooled to 0°C. Next, EtN(iPr)$_2$ (9.8 mL, 75.6 mmol, 1.2 eq) was added followed by o-toluidine (**S4**) (6.7 mL, 63.0 mmol, 1 eq) and the reaction was stirred at 0°C for 2 h. The reaction was poured into saturated aqueous ammonium chloride, extracted three times with CH_2Cl_2 and the solvent evaporated. The resulting solid was recrystallized from isopropanol and the mother liquor concentrated and purified by silica column chromatography to afford compound **S5** (14.3 g, 84%) as a tan solid.

TLC R_f: 0.70 (80/20 Hexanes/Ethyl Acetate)

^{1}H NMR (500 MHz, $CDCl_3$) δ 8.20 (s, 1H), 7.99 (d, J = 8.2 Hz, 1H), 7.57 (s, 1H), 7.43 – 7.33 (m, 5H), 7.23 (t, J = 6.9 Hz, 1H), 7.19 (d, J = 7.7 Hz, 1H), 7.08 (td, J = 7.5, 1.3 Hz, 1H), 5.25 (s, 2H), 2.27 (s, 3H).

^{13}C NMR (125 MHz, $CDCl_3$) δ 159.4, 143.9, 136.2, 135.1, 130.6, 128.8, 128.8, 128.7, 128.2, 127.1, 125.2, 122.0, 77.9, 17.6.

HRMS (ESI) calculated for $C_{16}H_{17}N_2O_2$ ($[M+H]^+$) 269.1290, found 269.1291.

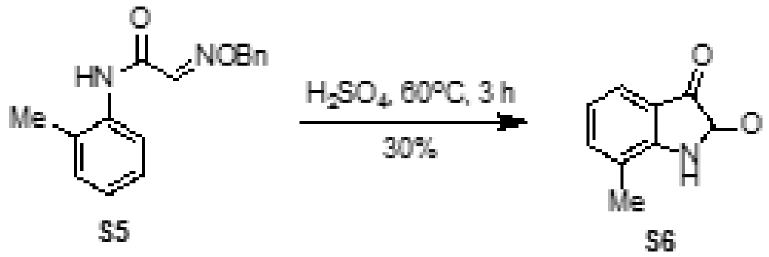

Compound S6: To a 250 mL round bottom flask was added sulfuric acid (125 mL) and the solution was heated to 60ºC. Compound **S5** (14.3 g, 53.2 mmol, 1 eq) was added in small batches over 3 h. After 3 h, the solution was added to crushed ice and stirred at room temperature for 30 min. The solution was extracted three times with ethyl acetate, dried through sodium sulfate, and evaporated. The resulting red/rust colored solid **S6** (2.61 g, 30%) was found to be poorly soluble in all solvents except DMSO, and identical in all respects with the known compound [1].

TLC R_f: 0.40 (60/40 Hexanes/Ethyl Acetate)

^{1}H NMR (500 MHz, d_6-DMSO) δ 11.07 (s, 1H), 7.41 (d, J = 7.6 Hz, 1H), 7.32 (d, J = 7.4 Hz, 1H), 6.97 (t, J = 7.5 Hz, 1H), 2.17 (s, 3H).

^{13}C NMR (125 MHz, d_6-DMSO) δ 184.8, 160.0, 149.3, 139.5, 122.7, 122.1, 121.6, 117.6, 15.5.

HRMS (ESI) calculated for $C_9H_8NO_2$ ($[M+H]^+$) 162.0555, found 162.0558.

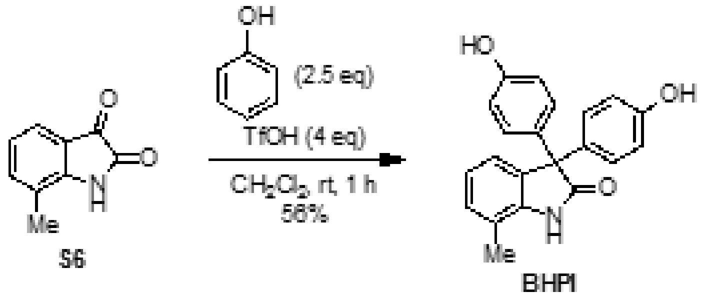

BHPI: To a 250 mL round bottom flask was added compound **S6** (2.60 g, 16.1 mmol, 1 eq), methylene chloride (80 mL) and phenol (3.79 g, 40.3 mmol, 2.5 eq). The solution was stirred at room temperature and trifluoromethanesulfonic acid (5.7 mL, 64.4 mmol, 4 eq) was added. The solution was stirred for 1 h and poured into aqueous sodium bicarbonate containing crushed ice. The aqueous solution was extracted three times with ethyl acetate, dried over sodium sulfate, concentrated and purified by silica column chromatography to give **BHPI** (3.0 g, 56%) as an off-white solid.

TLC R_f: 0.46 (40/60 Hexanes/Ethyl Acetate)

^{1}H NMR (500 MHz, CD_3OD) δ 7.06 – 6.99 (m, 5H), 6.97 – 6.90 (m, 2H), 6.73 – 6.66 (m, 4H), 2.30 (s, 3H).

13C NMR (125 MHz, CD_3OD) δ 183.1, 157.7, 140.7, 135.9, 134.4, 130.6, 130.3, 124.5, 123.5, 121.0, 116.0, 63.4, 16.8.

HRMS (ESI) calculated for $C_{21}H_{18}NO_3$ ($[M+H]^+$) 332.1287, found 332.1291.

LCMS:

tR = 6.52 min, ramp 95% A (water with 0.1% formic acid)/5% B (acetonitrile with 0.1% formic acid) to 70% A/30% B over 3 min, then ramp to 5% A/95% B over 3 min. Hold 5%A/95% B for 5 min.

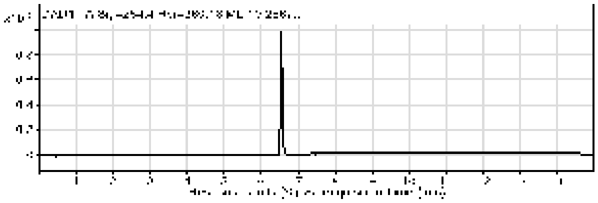

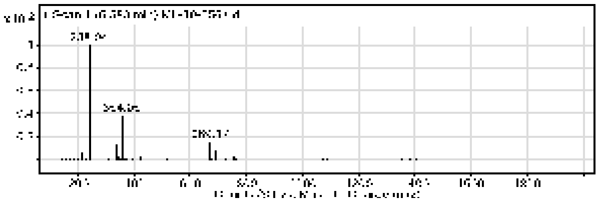

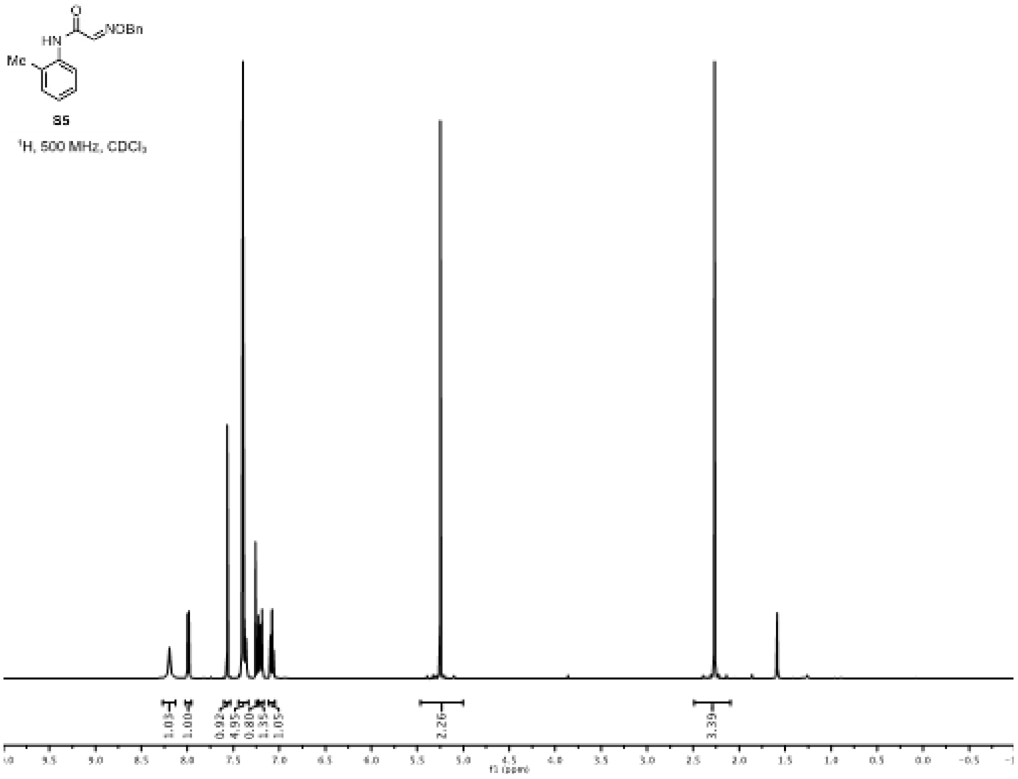
NOBn
HN
Me
S5
¹H, 500 MHz, CDCl3
1.03
1.00
0.92
4.95
0.80
1.35
1.05
2.26
3.39
f1 (ppm)

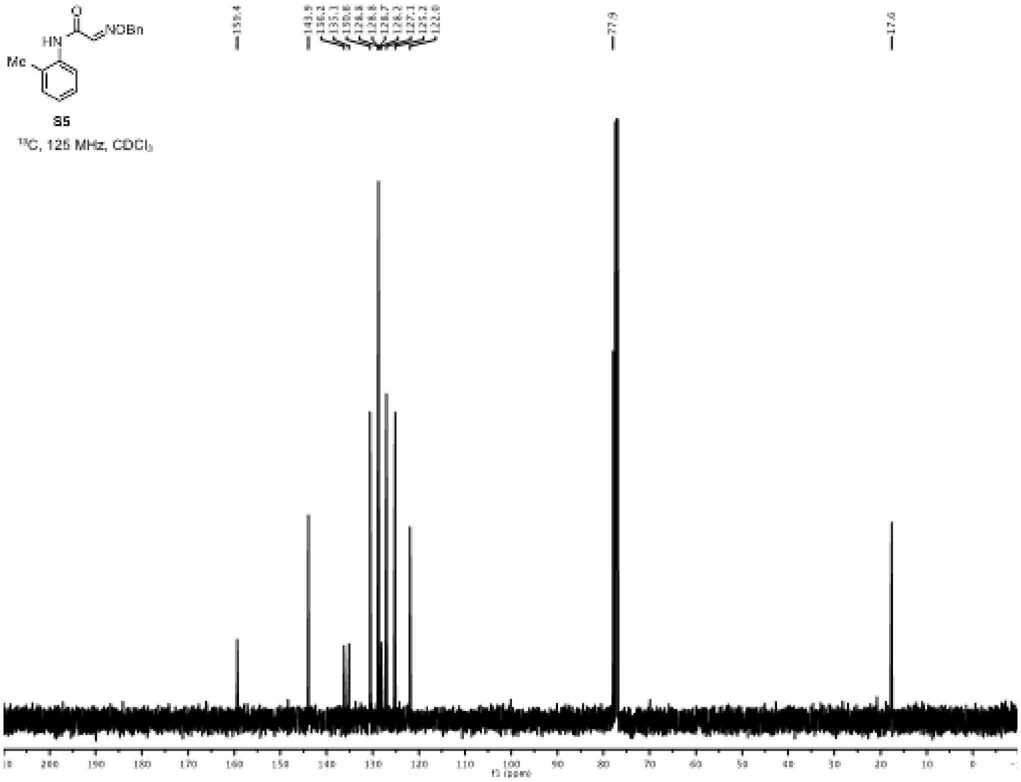
NOBn
HN
Me
S5
¹³C, 125 MHz, CDCl3
159.4
143.9
136.2
135.1
130.6
128.8
128.8
128.7
128.2
127.1
125.1
122.0
77.9
17.0
f1 (ppm)

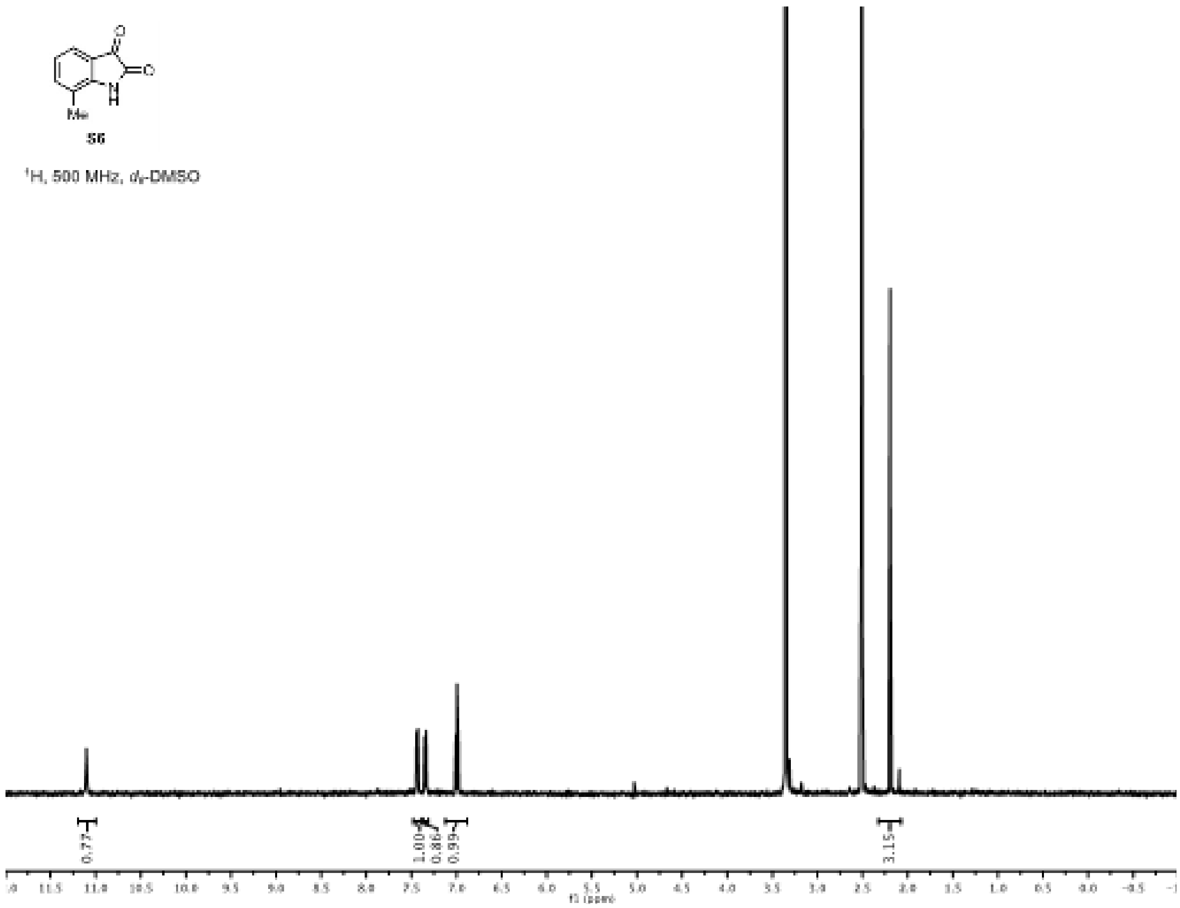
S6
¹H, 500 MHz, d6-DMSO
0.77
1.00
0.86
0.99
3.15
f1 (ppm)

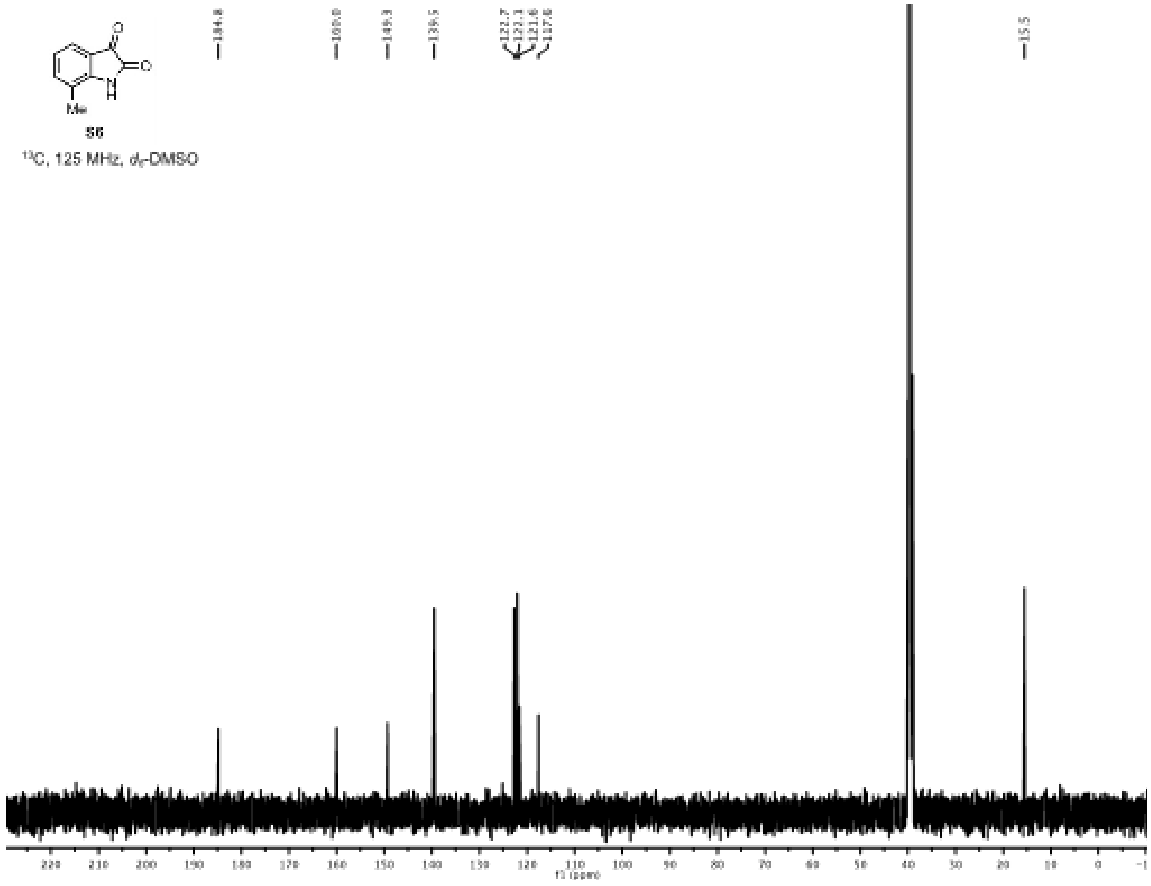
S6
¹³C, 125 MHz, d6-DMSO
184.8
160.0
149.3
139.5
122.7
122.1
121.6
117.6
15.5
f1 (ppm)

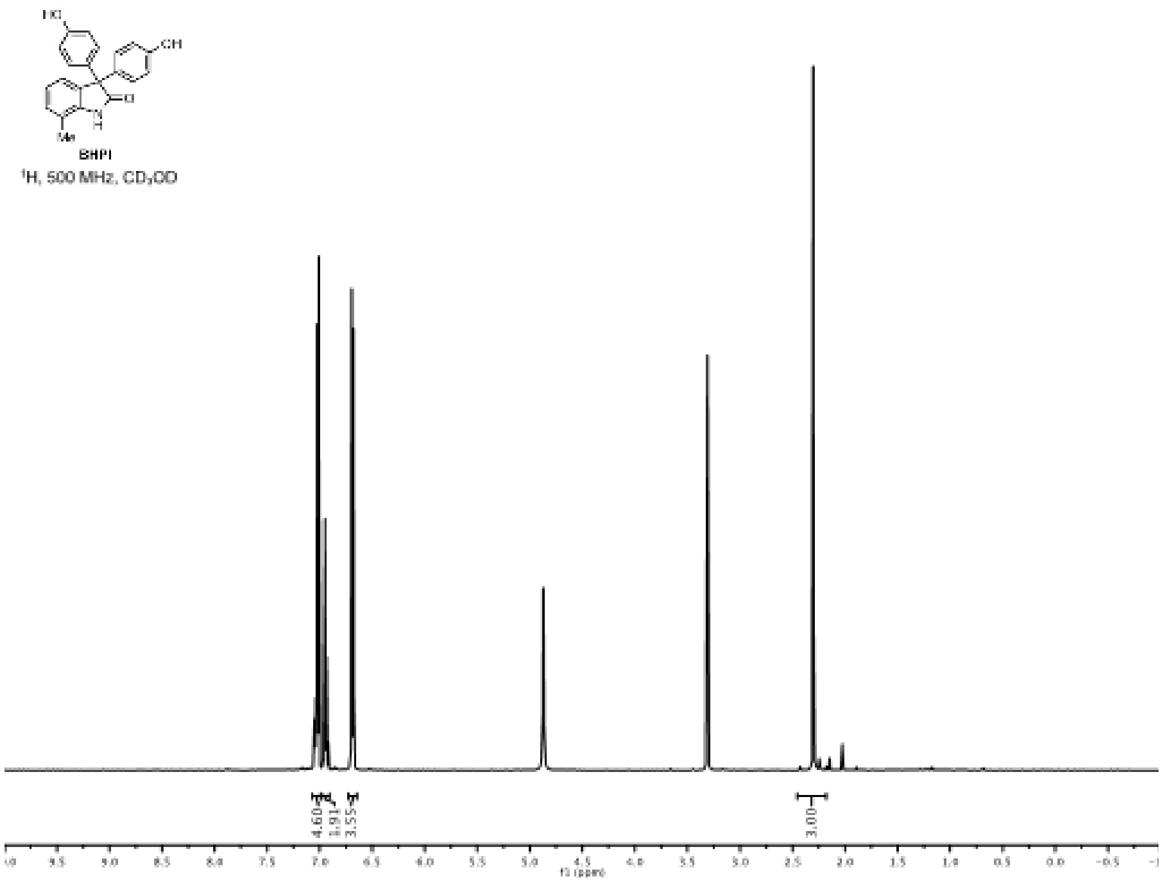
BHPI
1H, 500 MHz, CD3OD
4.60
1.91
3.55
3.00
f1 (ppm)

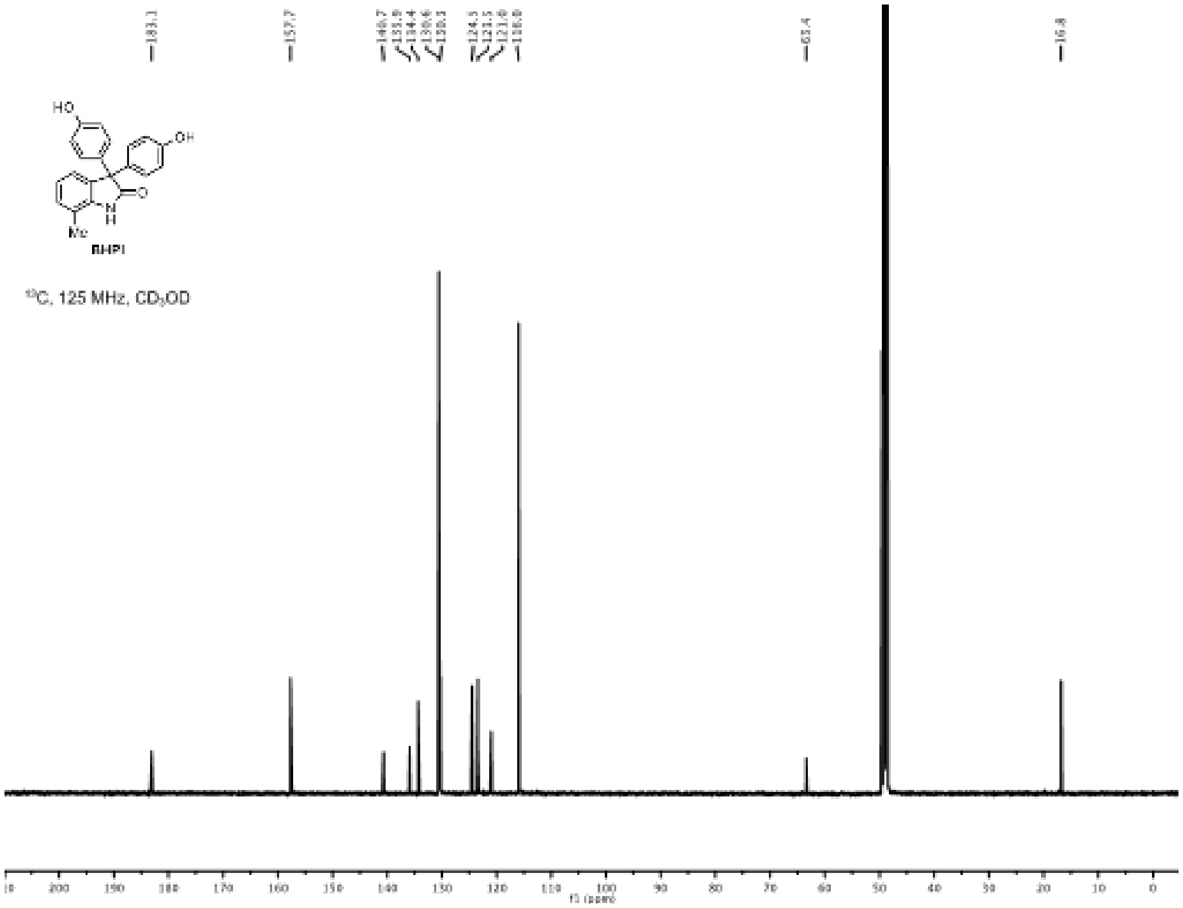
183.1
157.7
140.7
63.4
16.8
HO
OH
BHPI
13C, 125 MHz, CD3OD
f1 (ppm)

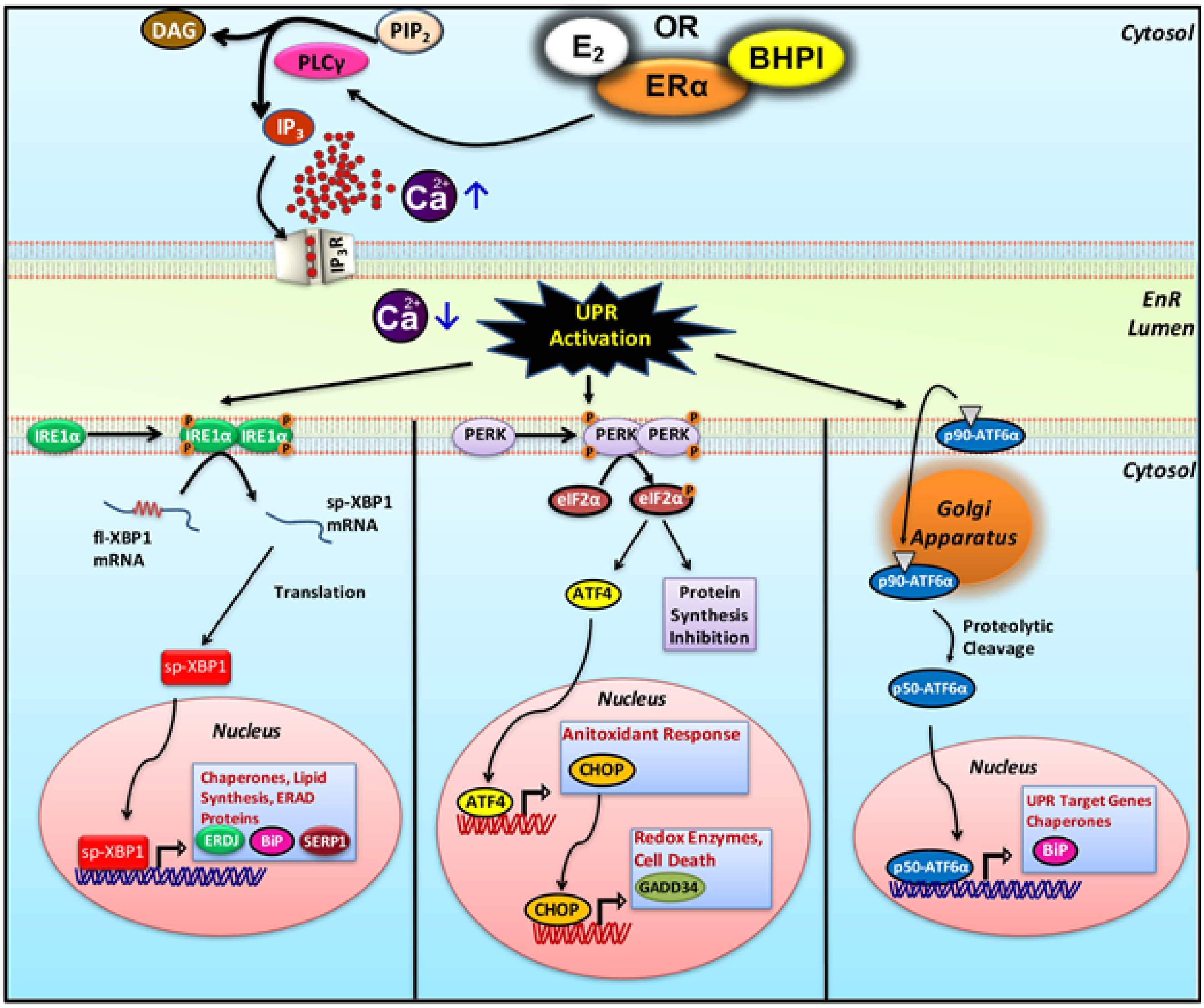

Supplementary Figure 1: Estrogen or BHPI mediated Ca^{2+} dependent UPR activation.

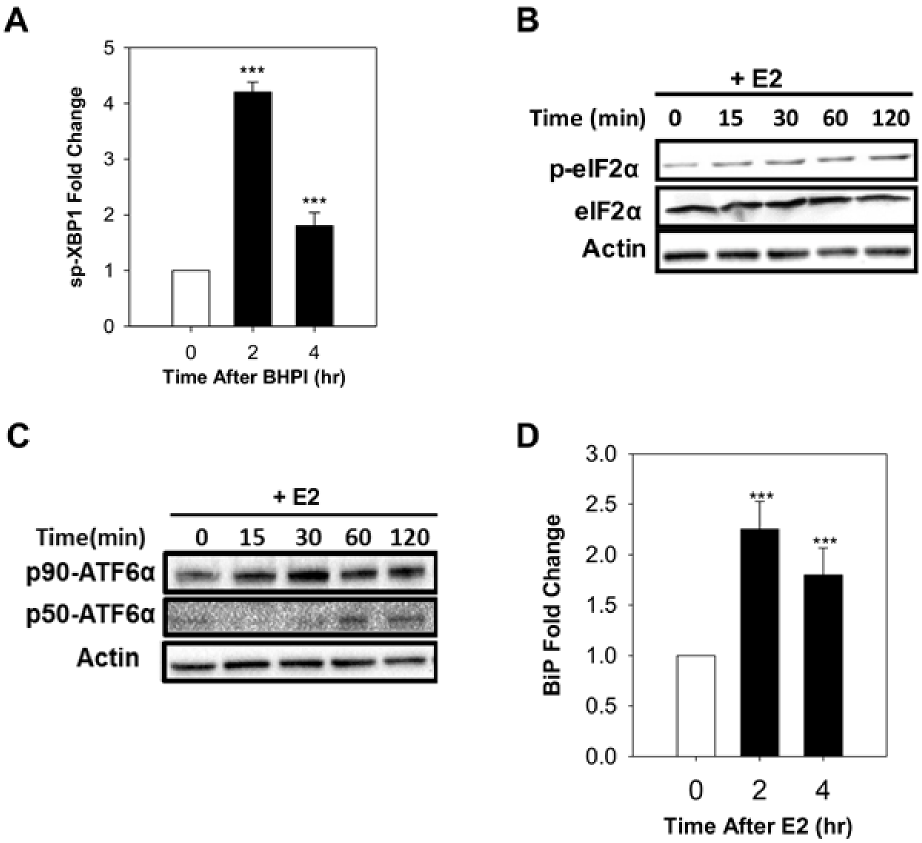

Supplementary Figure 2: Estrogen weakly activates the three arms of the UPR in PEO-4 cells. A. qRT-PCR analysis shows increasing sp-XBP1 mRNA after treatment with the 17β-estradiol (E_2). **B.** Western blot showing phosphorylated eIF2α and total eIF2α proteins. **C.** Effect of estrogen on the level of full-length (p90-ATF6α) and cleaved p50-ATF6α. **D.** qRT-PCT quantitation of BiP mRNA at indicated time points (n = 3). The concentration of E_2 is 10 nM. ***$P < 0.001$.

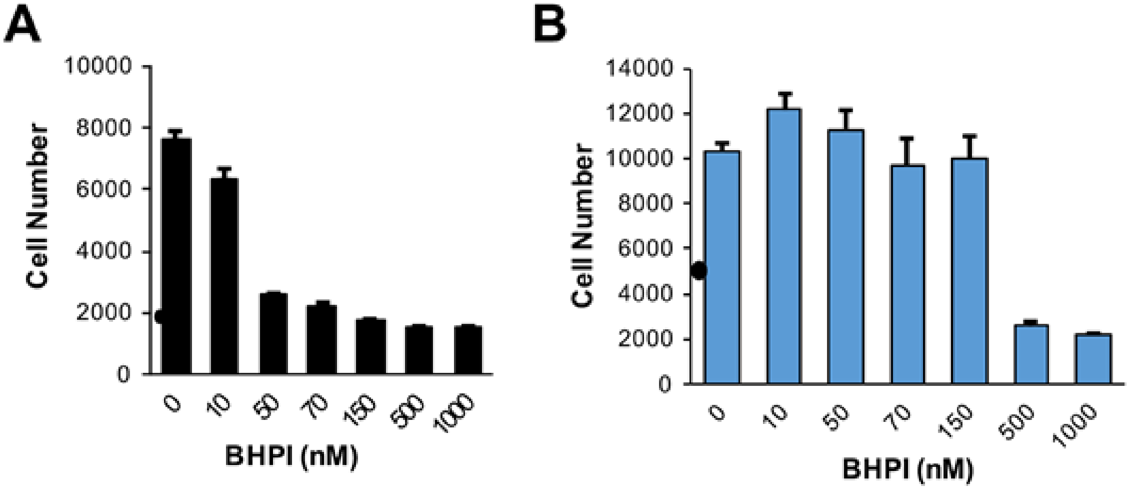

Supplementary Figure 3: BHPI blocks proliferation of MCF-7 and doxorubicin resistant MCF-7 ($MCF\text{-}7_{dox}$) breast cancer cells. A., **B.** MTS assays showing the effect of BHPI on cell proliferation. "•" on each graph denotes the number of cells at the start of the experiment. The cells were grown in medium containing 10 nM E_2 and the indicated concentrations of BHPI (n = 6). Data is the mean ± SEM.

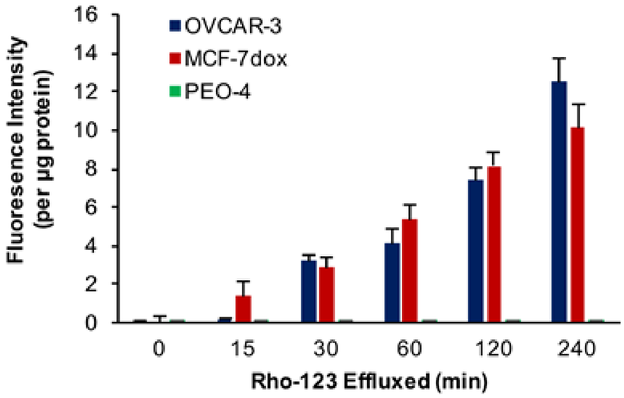

Supplementary Figure 4: MDR1 overexpressing cells exhibit higher MDR1 efflux activity than MDR1 negative cells. For each cell line, the Rho-123 fluorescence activity in the medium is quantitated at the indicated times ($n = 6$). Data is the mean ± SEM.

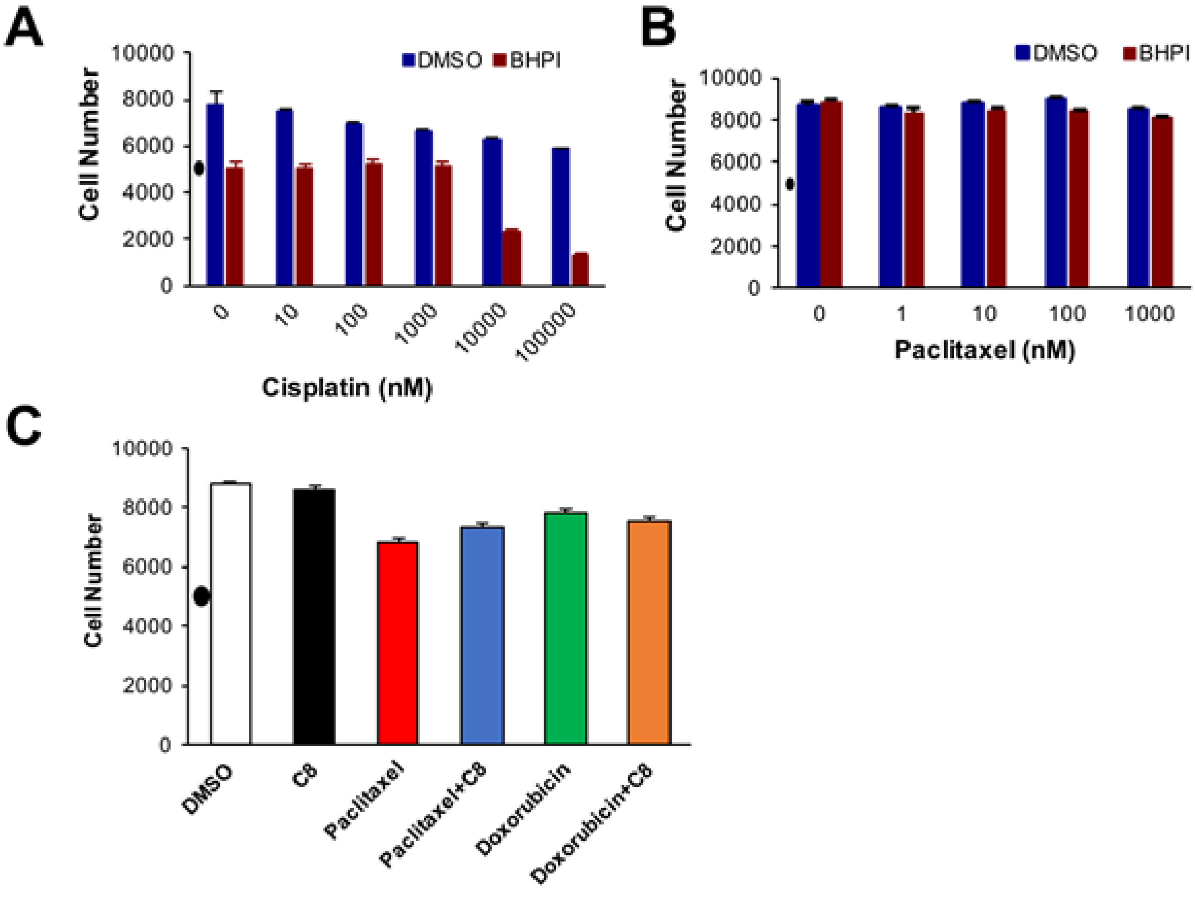

Supplementary Figure 5: BHPI does not restore cisplatin sensitivity in OVCAR-3 cells, does not restore paclitaxel sensitivity in MDR1 overexpressing ERα negative cells, and exhibits structure specificity. MTS assays showing the effect of BHPI on cisplatin sensitivity in ERα positive OVCAR-3 cells **A.** and on paclitaxel sensitivity in ERα negative NIH/ADRes cells **B.** ($n = 6$). **C.** The effect of an inactive close structural relative of BHPI compound 8 (C8) on OVCAR-3 cell proliferation was evaluated with either DMSO vehicle and together with paclitaxel or doxorubicin in ($n = 6$). "•" on each graph denotes the number of cells at the start of the experiment. The concentrations of BHPI, C8, paclitaxel and doxorubicin were 10 µM, 10 µM, 10 µM and 1 µM, respectively. Data is the mean ± SEM.

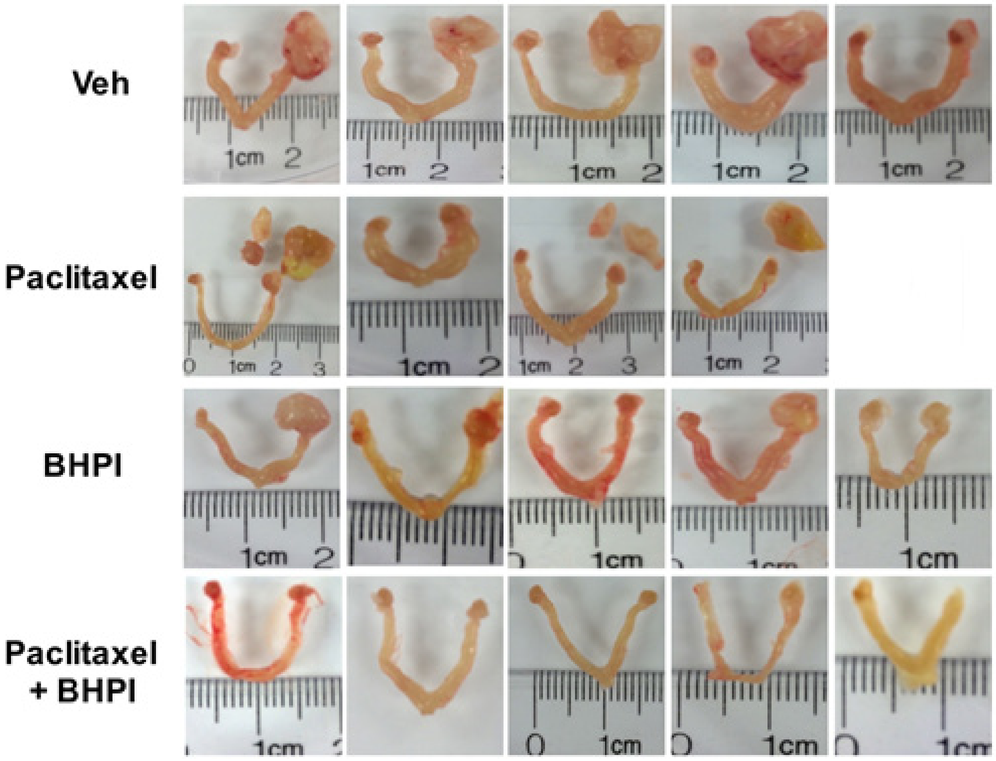

Supplementary Figure 6: BHPI plus paclitaxel eliminates orthotopic multidrug resistant OVCAR-3 tumors. Tumor images showing the size of OVCAR-3 tumors in vehicle (Veh) (*n* = 5), paclitaxel (*n* = 4, one image was lost), BHPI (*n* = 5) or paclitaxel plus BHPI (*n* = 5) treatment groups.

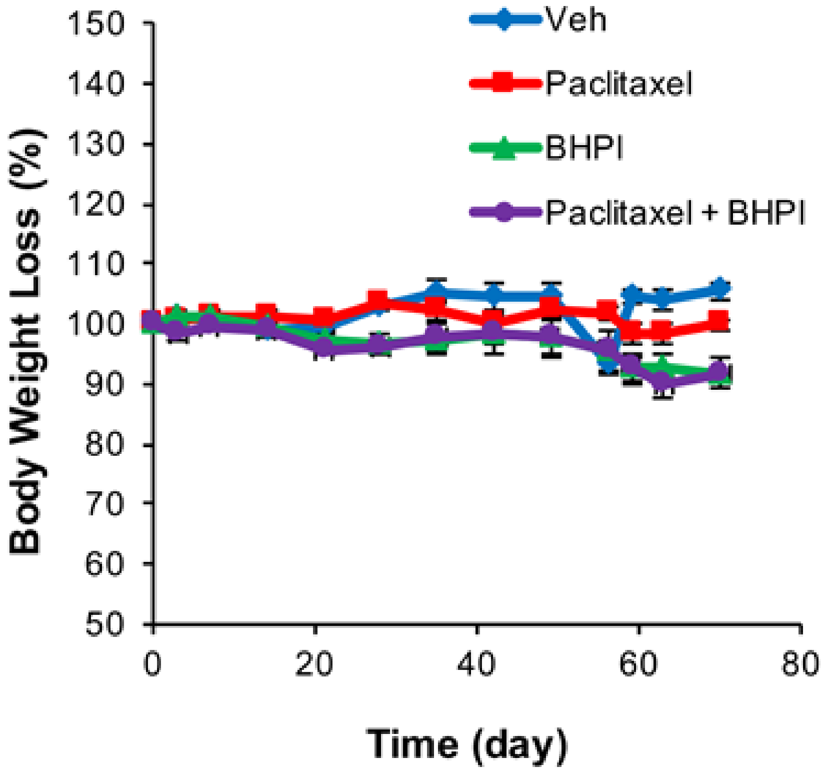

Supplementary Figure 7: BHPI and paclitaxel are well tolerated in mice. The body weight of mice was measured at the indicated time points. Data is the mean ± SEM (*n* =5).

Supplementary Movie 1: Effect of estrogen on cytosolic calcium levels in PEO-4 ovarian cancer cells. Cells were treated with 200 nM E_2 in the absence (0 mM $CaCl_2$) of extracellular calcium. The fluorescence signaling was monitored using Fluo-4 AM calcium dye.

For Supplementary Movie 1, please see the attached file

Supplementary Movie 2: Effect of BHPI on cytosolic calcium levels in PEO-4 ovarian cancer cells. Cells were treated with 10 µM BHPI in the absence (0 mM $CaCl_2$) of extracellular calcium. The fluorescence signaling was monitored using Fluo-4 AM calcium dye.

For Supplementary Movie 2, please see the attached file

REFERENCES

1. Montoya-Pelaez PJ, Uh YS, Lata C, Thompson MP, Lemieux RP and Crudden CM. The synthesis and resolution of 2,2'-, 4,4'-, and 6,6'-substituted chiral biphenyl derivatives for application in the preparation of chiral materials. J Org Chem. 2006; 71(16):5921-5929.
